# An integrated approach based on FDA adverse event reporting system, network pharmacology, molecular docking, and molecular dynamics simulation analysis to study the cardiac adverse reactions and mechanism of action of osimertinib

**DOI:** 10.3389/fphar.2025.1619517

**Published:** 2025-06-09

**Authors:** Wenjuan Wang, Hong Liu, Yongqing Wen, Yanhua Zhang, Xu Ma

**Affiliations:** Key Laboratory of Carcinogenesis and Translational Research (Ministry of Education), Department of Pharmacy, Peking University Cancer Hospital and Institute, Beijing, China

**Keywords:** osimertinib, adverse event reporting system, molecular docking, network pharmacology, molecular dynamics simulations, cardiac adverse reactions

## Abstract

**Objectives:**

This study investigates osimertinib-induced cardiac adverse reactions (CAR) using real-world FDA Adverse Event Reporting System (FAERS) data and explores molecular mechanisms via network pharmacology, molecular docking, and dynamics simulations.

**Methods:**

We analyzed osimertinib-related adverse events from Q4 2015 to Q4 2024 using FAERS data, applying reporting odds ratio (ROR) and Bayesian confidence propagation neural network (BCPNN) methods. Potential CAR targets were identified via PharmMapper, Swiss Target Prediction, and GeneCards. Protein-protein interaction (PPI) networks, gene ontology (GO), Kyoto Encyclopedia of Genes and Genomes (KEGG) pathway analyses, molecular docking, and dynamics simulations were performed.

**Results:**

Among 15,382 reports, 274 were CAR-related, including pericardial effusion, cardiomyopathy, and cardiac dysfunction (25.00% mortality). Key targets (AKT1, ESR1, EGFR, SRC, ALB, CASP3) and pathways (PI3K-Akt, Ras, MAPK, calcium, JAK-STAT, TNF) were identified. Molecular docking confirmed strong binding affinity with binding energies below −7.5 kJ/mol for key targets (AKT1: -9.9 kJ/mol; ALB: −8.4 kJ/mol). Molecular dynamics simulations (100 ns) demonstrated stable binding of osimertinib-AKT1/ALB complexes, with average RMSD values of 0.52 nm and 0.50 nm, respectively, and binding free energies of −44.63 kJ/mol (AKT1) and −42.92 kJ/mol (ALB).

**Conclusion:**

This study clarifies osimertinib-induced CAR mechanisms involving multi-target interactions and pathway dysregulation, aiding clinical safety and future research.

## 1 Introduction

Osimertinib represents the initial third-generation epidermal growth factor receptor (EGFR) kinase inhibitor designed for oral administration, with worldwide availability. It is mainly used to treat non-small cell lung cancer (NSCLC) caused by EGFR gene mutations, such as exon 19 deletions, exon 21 L858R substitutions, and T790M resistance mutations (Cross et al., 2014; Hochmair et al., 2019; Bertoli et al., 2022). Osimertinib was approved by the US Food and Drug Administration in November 2015, which later gained regulatory approval in Europe, Japan, and China in 2017. Clinical trial results suggest that the predominant adverse reactions linked to osimertinib use are rash, diarrhea, dry skin, paronychia, stomatitis, and anorexia (Mok et al., 2017). Several studies have indicated that osimertinib is linked to a greater frequency of severe cardiac adverse events when compared to conventional EGFR-TKIs. These events include acute myocardial infarction, angina pectoris, atrial fibrillation, cardiac arrest, heart failure, and arrhythmia. Notably, there are occasional cases of fatalities attributed to osimertinib-induced cardiotoxicity. In contrast, the incidence of serious adverse events in other systems is lower for osimertinib than for standard EGFR-TKIs (Soria et al., 2018; Zaborowska-Szmit et al., 2020; Bolzon et al., 2024). However, drug-related toxicity can lead to the temporary or premature discontinuation of anti-cancer treatment, which may result in an unfavorable prognosis. Therefore, a deeper understanding of osimertinib-induced cardiotoxicity and its underlying pathogenesis, along with early prevention, diagnosis, and intervention strategies, is crucial for enhancing the outcomes in patients with NSCLC.

Network pharmacology examines the interactions among drugs, targets, and diseases from an integrated and systematic viewpoint, employing complex network models to represent and analyze the pharmacological characteristics of research subjects. This method is particularly suitable for elucidating the multi-target effects of drugs (Deng et al., 2020; Zhang et al., 2022). Molecular docking technology predicts the interaction capabilities of ligands with target proteins at the molecular level, enabling computer-aided drug screening and prediction of ligand-receptor affinity. Based on the “lock-and-key principle,” molecular docking can further validate the core targets identified through network pharmacology (Jiao et al., 2021) The utilization of Molecular Dynamics Simulation (MD) in pharmaceutical research is gaining significant traction. By modeling the spatiotemporal dynamics of atoms and molecules, MD simulation offers valuable insights into the microscopic mechanisms underlying drug action, target recognition, drug design optimization, and safety assessment (Hu et al., 2023; Hu et al., 2024).

Therefore, this study retrospectively analyzed relevant data from the FAERS to identify signals of osimertinib-induced cardiac adverse reactions (CAR) and assess the associated risks in real-world applications. Furthermore, the study utilized network pharmacology, molecular docking and molecular dynamics techniques to identify the critical targets associated with osimertinib-induced CAR and investigate the corresponding molecular mechanisms. This approach aims to offer a reliable scientific foundation for ensuring the safe clinical application of osimertinib.

## 2 Materials methods

### 2.1 Analysis of adverse reactions

#### 2.1.1 Data sources

OpenVigil 2.1 (http://openvigil.sourceforge.net/) serves as an online platform for data mining and pharmacovigilance analysis, which has been widely utilized in pharmacovigilance research (Böhm et al., 2021). In this study, we retrieved data from the FAERS database using OpenVigil 2.1. The search period was set from the fourth quarter of 2015 to the fourth quarter 2024, with the target drug name limited to “Osimertinib”. We obtained ADR reports where osimertinib was listed as the primary suspect drug. We utilized the Medical Dictionary for Regulatory Activities (MedDRA; https://www.meddra.org/), created by the International Council for Harmonisation (ICH; https://www.ich.org/), to extract reports of ADRs related to the cardiac system (complete list of PTs in Supplementary Table S1). The top five most frequently reported cardiac ADRs were included in the analysis. Additionally, demographic information such as age, gender, country or region, and outcome details were also extracted.

#### 2.1.2 Signal detection method

This study employed the Reporting Odds Ratio (ROR) method and the Bayesian Confidence Propagation Neural Network (BCPNN) method for data mining. The ROR method relies on a fourfold table to analyze proportion imbalance (Chuma et al., 2022). While it exhibits high sensitivity, it has relatively low specificity, potentially leading to false positives. In contrast, the BCPNN method combines Bayesian logic with a neural network framework, providing more reliable outcomes and enhanced specificity (Bate et al., 1998; Luo et al., 2024). For signal detection, the ROR method generates a positive signal when the number of cases is greater than or equal to 3 and the lower limit of the 95% confidence interval exceeds 1. For the BCPNN method, a positive signal is generated when the number of cases is greater than or equal to 3 and the lower bound of the 95% confidence interval (IC025) exceeds 0. Detailed formulas and thresholds for both methods are provided in Supplementary Tables S2, S3 Combining these two methods mitigates potential biases from using a single algorithm and addresses issues of low specificity and high false positives that may arise from relying on one approach alone. The emergence of ADR signals suggests a statistical link between the drug and the adverse event. A stronger signal implies a more significant relationship between them. The classification criteria for signal strength are as follows: for the ROR method, 1 < ROR - 1.96SE < 50 is a weak signal; 50 ≤ ROR - 1.96SE < 1,000 is a moderate strength signal; ROR - 1.96SE ≥ 1,000 is a strong signal. For the BCPNN method, 0 < IC - 2SD ≤ 1.5 is a weak signal; 1.5 < IC - 2SD ≤ 3.0 is a moderate strength signal; IC - 2SD > 3.0 is a strong signal (Guan et al., 2022).

### 2.2 Network pharmacology analysis

#### 2.2.1 Collection of targets of osimertinib and CAR

The SMILES notation of osimertinib was obtained from the PubChem database (https://pubchem.ncbi.nlm.nih.gov/). The target proteins associated with osimertinib were identified using the Swiss Target Prediction database (http://www.swisstargetprediction.ch/), the PharmMapper database (http://lilab-ecust.cn/pharmmapper/), and the UniProt database (https://www.uniprot.org/). Using the keywords “pericardial effusion”, “cardiomyopathy”, “cardiotoxicity”, “acute cardiac failure”, and “cardiac dysfunction”, we performed a search in the GeneCards database (https://www.genecards.org/) with a screening criterion of “correlation score ≥10” to identify CAR-related targets. Subsequently, by calculating the median and excluding genes with lower correlation scores, the results from these five searches were integrated. Duplicate entries were removed to generate a final list of potential CAR-related targets. The intersection of osimertinib targets and CAR targets was considered as the common targets, and a Venn diagram was generated using the Venny tool (http://bioinformatics.psb.ugent.be/webtools/venn/).

#### 2.2.2 Signal pathways and functional enrichment analysis

The Kyoto Encyclopedia of Genes and Genomes (KEGG) signal pathway analysis and Gene Ontology (GO) function analysis were conducted on key targets using the Metascape database (https://www.metascape.org/). A threshold value of P < 0.01 was set, leading biological processes were identified, and a bubble map for KEGG enrichment analysis was generated. Based on this, the key signaling pathways, cellular components (CC), molecular functions (MF), and biological processes (BP) were thoroughly investigated and comparatively analyzed to elucidate the potential underlying mechanism by which osimertinib leads to major adverse cardiac reactions.

#### 2.2.3 Network construction

The potential targets linked to the major CAR induced by osimertinib were investigated using the STRING database (https://string-db.org/). Initially, the species “*Homo sapiens*” was specified, and a threshold of ≥0.9 for the “highest confidence” score was set. Following this, protein-protein interaction (PPI) network analysis was carried out. The resulting TSV format data was then imported into Cytoscape 3.7.2 software (Shannon et al., 2003) to generate the interaction map between proteins. To systematically identify key core targets within the PPI network, a comprehensive network topology analysis method was employed. Initially, using the Network Analyzer function in Cytoscape 3.7.2, topological parameters of each node were calculated, and the top 10 core targets were selected based on their degree values. To further validate the reliability of these findings, the cytoHubba plugin in Cytoscape 3.7.2 was utilized for multi-algorithm cross-validation, with particular emphasis on the Maximal Clique Centrality (MCC) algorithm due to its superior ability to recognize biologically relevant hubs. Using Cytoscape 3.7.2, the network connecting osimertinib’s compounds, targets, diseases, and pathways related to CAR was also established.

### 2.3 Molecular docking

The compounds and core targets that played significant roles in the network were chosen as ligands and receptors for molecular docking analysis. The SMILES notation of the ligand small molecule was retrieved from the PubChem database (https://pubchem.ncbi.nlm.nih.gov/). Using Chimera 1.16 software (Pettersen et al., 2004), the SMILES notation of the small molecule was imported to generate its 3D structure. Energy minimization was then performed on the ligand, and the optimized structure was saved as a pdb format file. The pdb file of the target protein was downloaded from the PDB database (http://www.rcsb.org/pdb/), with the source selected as *Homo sapiens*. Structural proteins and their codes are detailed in Table 1. Chimera1.16 software, the downloaded receptor protein in pdb format was processed by removing any bound ligands and ions, and the cleaned protein structure was saved as a pdb format file. Molecular docking simulations were conducted using Chimera, with 50 independent runs performed. During the construction of the docking box, it was centered on the receptor protein, ensuring that the docking box fully encompassed the receptor while positioning the ligand outside the box. Binding activity between the receptor and ligand was assessed based on binding energy values, and receptor-ligand pairs with superior binding affinities were identified. The visualization of the 3D complex was performed using PyMOL 2.6.0 (Alexander et al., 2011), while the representation of the 2D complex was carried out using Discovery Studio Visualizer software (Aminu et al., 2021).

**TABLE 1:** Details of the protein targets in the PDB database.

Targets	PDB ID	Method	Resolution (Å)	R-Value free	R-Value work	R-Value observed
AKT1	4EJN	X-RAY DIFFRACTION	2.19	0.276	0.237	0.239
ALB	3B9M	X-RAY DIFFRACTION	2.7	0.316	0.242	–
MMP9	5CUH	X-RAY DIFFRACTION	1.83	0.214	0.173	0.175
CASP3	3KJF	X-RAY DIFFRACTION	2	0.206	0.181	0.183
EGFR	3QKQ	X-RAY DIFFRACTION	2.2	0.254	0.196	0.199
ESR1	4EJN	X-RAY DIFFRACTION	2.19	0.276	0.237	0.239
PPARG	6MD4	X-RAY DIFFRACTION	2.24	0.282	0.24	0.242
IGF1	3D94	X-RAY DIFFRACTION	2.3	0.236	0.198	0.2
IL2	1M48	X-RAY DIFFRACTION	1.95	0.269	0.197	0.2
SRC	3UQG	X-RAY DIFFRACTION	2.2	0.259	0.229	0.23

### 2.4 Molecular dynamics simulations

Based on the molecular docking results, this study identified two targets that exhibited favorable interactions with osimertinib. To further investigate the dynamic characteristics of the docked complexes, 100 ns molecular dynamics (MD) simulations were performed for the osimertinib-AKT1 and osimertinib-ALB complexes. GROMACS 2022.3 software was employed for molecular dynamics (MD) simulations (Van Der Spoel et al., 2005; Abraham et al., 2015). For small molecule preprocessing, AmberTools22 (Sousa da Silva and Vranken, 2012) was utilized to assign the GAFF force field to small molecules, while Gaussian 16W was used for hydrogenation and RESP potential calculation. The resulting potential data were incorporated into the topology file of the MD system. Simulations were conducted under a constant temperature of 300 K and atmospheric pressure (1 bar). The Amber99sb-ildn (Hornak et al., 2006) force field was adopted, with TIP3P water molecules serving as the solvent. The total charge of the simulation system was neutralized by adding an appropriate number of Na^+^ ions. Energy minimization was performed using the steepest descent method, followed by 100,000-step equilibration in the isothermal-isochoric ensemble (NVT) and isothermal-isobaric ensemble (NPT), with a coupling constant of 0.1 ps and a duration of 100 ps. Subsequently, free MD simulations were carried out, consisting of 5,000,000 steps with a step size of 2 fs, corresponding to a total simulation time of 100 ns (Bussi et al., 2007). Upon completion of the simulation, the software’s built-in tools were used to analyze the trajectory. Root-mean-square deviation (RMSD), root-mean-square fluctuation (RMSF), radius of gyration for each amino acid trajectory, along with free energy calculations (MMGBSA) and free energy landscapes, were computed (Liu M. et al., 2024).

## 3 Results

### 3.1 Analysis of adverse reactions

A total of 33,744 ADR reports with osimertinib as the primary suspect drug were retrieved from the FAERS database. After screening based on the ADR signal determination criteria, 15,382 reports were included for analysis. Among these, 274 reports pertained to cardiovascular system ADRs. The top five CAR were pericardial effusion (59 cases), cardiomyopathy (47 cases), cardiac toxicity (42 cases), acute heart failure (25 cases), and cardiac dysfunction (24 cases), as detailed in Table 2. The demographic characteristics and outcomes of osimertinib-induced cardiac toxicity are presented in Table 3. Pericardial effusion was the most common CAR, accounting for 0.38% of all ADRs and 21.53% of all CAR reports. Among all CAR reports, the reporting proportion was significantly higher in females compared to males. The age distribution was predominantly in the 60–80 age group. The report source analysis indicated that the highest number of major cardiac adverse reaction reports originated from Asia, followed by the Americas. The mortality rates associated with osimertinib-induced cardiotoxicity are as follows: cardiac dysfunction (25.00%), acute heart failure (20.00%), pericardial effusion (16.95%), cardiomyopathy (12.77%), and general cardiac toxicity (9.52%). Among patients with cardiomyopathy, the highest disability rate was observed at 2.13%. In patients with cardiac dysfunction, the proportion of life-threatening events was the highest, reaching 16.67%.

**TABLE 2:** Reporting of adverse events in the cardiac disorders from 2015 to 2024.

PT	Reports number	Percentage/% (Cardiac ADR)	Percentage/% (full database)	ROR (95%CI)
Pericardial effusion	59	21.53	0.38	3.90 (3.02∼5.04)
Cardiomyopathy	47	17.15	0.31	5.31 (3.98∼7.08)
Cardiotoxicity	42	15.33	0.27	5.03 (3.71∼6.83)
Cardiac failure acute	25	9.12	0.16	5.15 (3.47∼7.64)
Cardiac dysfunction	24	8.76	0.16	6.02 (4.02∼9.00)

**TABLE 3:** Population characteristics and outcomes of adverse cardiac reactions caused by osimertinib.

Characteristics	Pericardial effusion/n (%)	Cardiomyopathy/n (%)	Cardiotoxicity/n (%)	Cardiac failure acute/n (%)	Cardiac dysfunction/n (%)
Total number of reported ADR	59 (100.00)	47 (100.00)	42 (100.00)	25 (100.00)	24 (100.00)
Sex					
Female	35 (59.32)	26 (55.32)	21 (50.00)	14 (56.00)	11 (45.83)
Male	19 (32.20)	9 (19.15)	13 (30.95)	11 (44.00)	5 (20.83)
Unknown or missing	5 (8.47)	12 (25.53)	8 (19.05)	0 (0.00)	8 (33.33)
Age					
<60	9 (15.25)	1 (2.13)	3 (7.14)	1 (4.00)	1 (4.17)
60∼80	21 (35.59)	22 (46.81)	17 (40.48)	16 (64.00)	11 (45.83)
>80	3 (5.08)	6 (12.77)	6 (14.29)	7 (28.00)	3 (12.50)
Unknown or missing	26 (44.07)	18 (38.30)	16 (38.10)	1 (4.00)	9 (37.50)
Outcome					
Death	10 (16.95)	6 (12.77)	4 (9.52)	5 (20.00)	6 (25.00)
Disabled	1 (1.69)	1 (2.13)	0 (0.00)	0 (0.00)	0 (0.00)
Life-threatening	8 (13.56)	4 (8.51)	3 (7.14)	3 (12.00)	4 (16.67)
Hospitalization	34 (57.63)	17 (36.17)	9 (21.43)	17 (68.00)	9 (37.50)
Other outcomes	6 (10.17)	19 (40.43)	26 (61.90)	0 (0.00)	5 (20.83)
Outcome counts by year received					
2020	8 (13.56)	5 (10.64)	7 (16.67)	2 (8.00)	2 (8.33)
2021	5 (8.47)	4 (8.51)	6 (14.29)	3 (12.00)	1 (4.17)
2022	5 (8.47)	5 (10.64)	6 (14.29)	3 (12.00)	3 (12.50)
2023	10 (16.95)	8 (17.02)	11 (26.19)	2 (8.00)	3 (12.50)
2024					
Region of ADR					
Asia	18 (30.51)	13 (27.66)	22 (52.38)	10 (40.00)	12 (50.00)
Americas	10 (16.95)	15 (31.91)	8 (19.05)	2 (8.00)	1 (4.17)
Europe	8 (13.56)	4 (8.51)	9 (21.43)	1 (4.00)	0 (0.00)
Australia	0 (0.00)	0 (0.00)	0 (0.00)	0 (0.00)	1 (4.17)

According to disproportionality signal analysis, compared with all other drugs in the FAERS database, osimertinib exhibited significantly elevated ROR values for several CAR: pericardial effusion (ROR = 3.9, 95% CI: 3.02–5.04), cardiomyopathy (ROR = 5.31, 95% CI: 3.98–7.08), cardiac toxicity (ROR = 5.03, 95% CI: 3.71–6.83), acute heart failure (ROR = 5.15, 95% CI: 3.47–7.64), and cardiac dysfunction (ROR = 6.02, 95% CI: 4.02–9.00). Among the reactions with elevated ROR, the highest reported mortality rate was associated with cardiac dysfunction, at 25%. These findings are summarized in Tables 2, 3.

### 3.2 Network pharmacology analysis

#### 3.2.1 Screening of intersection-targets

**FIGURE 1:**
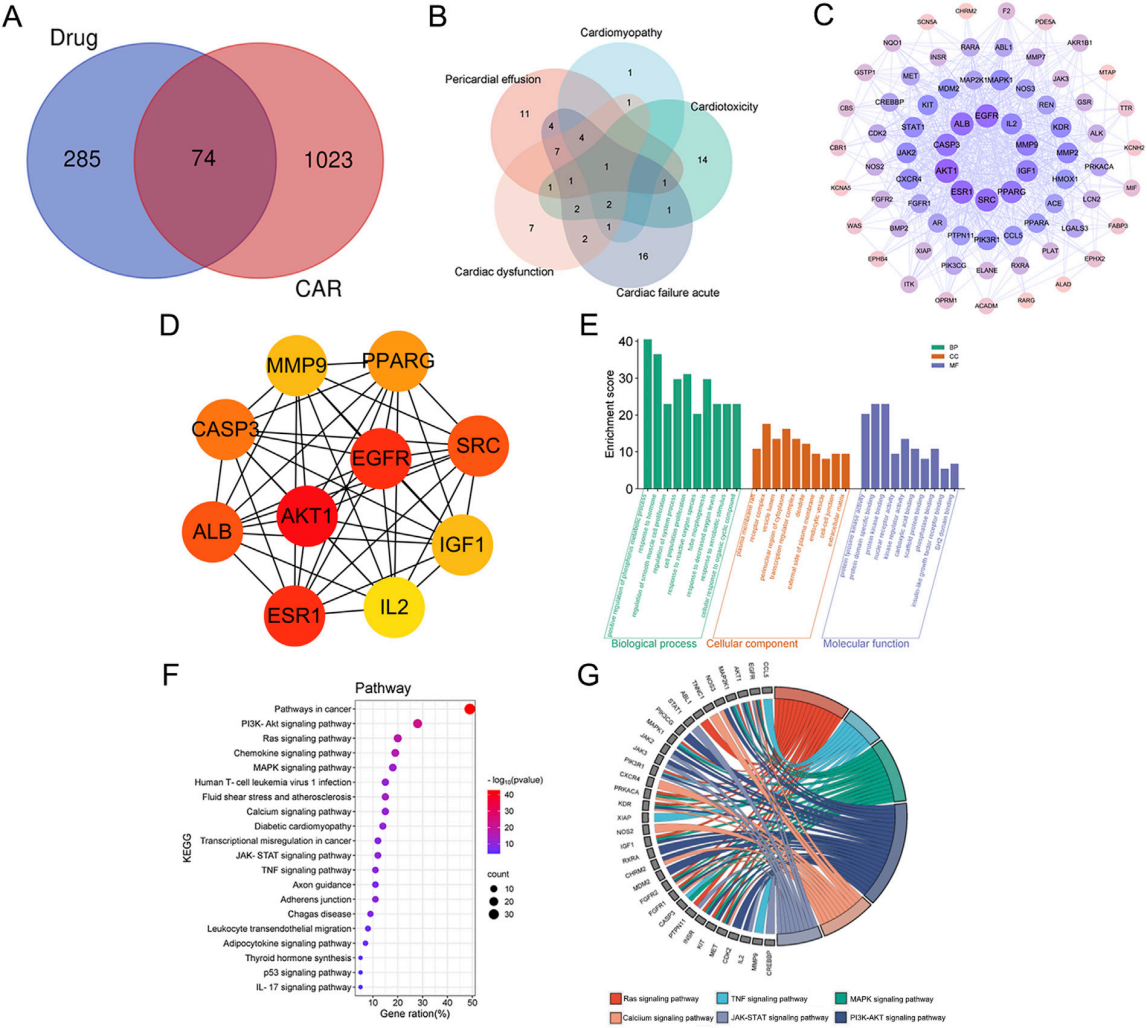
The potential pharmacological mechanisms of osimertinib-induced cardiac adverse reactions (CAR). **(A)** Venn diagram of intersectional genes between osimertinib and CAR; **(B)** Vene diagram of gene targets of severe CAR; **(C)** PPI network of the common targets; **(D)** Core targets; **(E)** Gene Ontology (GO) enrichment analysis; **(F)** Kyoto Encyclopedia of Genes and Genomes (KEGG) enrichment analysis; **(G)** The chord diagram of the key signaling pathways associated with CAR.

The potential targets for osimertinib (359) were retrieved from multiple databases, including Swiss Target Prediction database, PharmMapper database, and the UniProt database. A total of 1,097 targets of CAR from GeneCards database. Mapping the targets of osimertinib with adverse reactions of the heart targets through Venn diagram, 74 intersection targets were obtained (Figure 1A). The potential targets are presented in Supplementary Table S4 Additionally, we constructed a map of these target genes and discovered that there are intersections among these targets (Figure 1B). This suggests that various adverse cardiac reactions may act on the same genes.

#### 3.2.2 Construction of PPI network

The 74 potential targets were uploaded to the String database for constructing the PPI network interaction. Initially, using the Network Analyzer function in Cytoscape 3.7.2, we calculated the topological parameters of each node and selected the top 10 core targets (AKT1, EGFR, SRC, ESR1, ALB, CASP3, MMP9, IGF1, IL2, and PPARG) based on their degree values. The visualization results are presented in Figure 1C. To further validate the reliability of these findings, we employed the cytoHubba plugin for multi-algorithm cross-validation, with particular emphasis on the MCC algorithm due to its superior ability to identify biologically relevant hubs. As shown in Figure 1D, the top 10 candidate targets identified by MCC scores exhibited high consistency with those determined by other centrality metrics, including degree centrality, betweenness centrality, and closeness centrality. These selected core targets are likely to be the key targets underlying CAR induced by osimertinib.

#### 3.2.3 GO and KEGG enrichment analysis

We carried out a functional enrichment analysis using GO analysis to investigate the biological functions of the potential targets and their intersection with CAR. The Metascape database was utilized to perform GO enrichment analysis on 74 common targets. The findings revealed that a total of 631 MF items, 3902 BP items, and 305 CC items were collected in the dataset. Due to the extensive number of items, the Metascape platform employs Kappa scores as the similarity parameter. Hierarchical clustering is then performed on items with a similarity greater than 0.3, and within each cluster, the most statistically significant item represents the entire cluster. Figure 1E showcases the top 10 BP, MF, and CC items along with their corresponding enrichment scores. The targets in biological processes were mainly for the positive regulation of phosphorus metabolic process, response to hormone, and regulation of smooth muscle cell proliferation. The molecular function mainly involves protein tyrosine kinase activity, protein domain specific binding, protein kinase binding. The cellular component includes plasma membrane raft, receptor complex, vesicle lumen. The results indicate that osimertinib plays a regulatory role in the biological processes associated with CAR.

In order to investigate the signaling pathways associated with the regulatory targets of CAR induced by osimertinib, KEGG enrichment analysis was conducted on potential targets using the Metascape database. A total of 239 KEGG signaling pathways were identified, respectively. Hierarchical clustering analysis was carried out, and bubble maps representing the top 20 signaling pathways were generated using a bioinformatics platform (https://www.bioinformatics.com.cn/) (Figure 1F). Through literature review and KEGG enrichment analysis (P < 0.01), we identified that the potential targets were primarily enriched in signaling pathways associated with adverse cardiac reactions, including: PI3K-Akt signaling pathway (Ghafouri-Fard et al., 2022; Liu et al., 2017), which regulates cardiomyocyte survival and apoptosis. Ras and MAPK signaling pathways (Heineke and Molkentin, 2006; Zhou et al., 2022), implicated in myocardial hypertrophy and apoptosis. Calcium signaling pathway (Metwally et al., 2020), critical for cardiac contraction and relaxation. JAK-STAT signaling pathway (Chen et al., 2023; Magaye et al., 2020), linked to inflammation and fibrosis. TNF signaling pathway (Yamaguchi et al., 2022), associated with NLRP3-mediated cardiac fibrosis. These pathways were selected based on their established roles in drug-induced cardiotoxicity and their statistical significance in KEGG analysis. Figure 1G illustrates the enrichment relationships between key signaling pathways associated with CAR and their corresponding targets.

#### 3.2.4 Construction of drug-targets-CAR-pathways network

**FIGURE 2:**
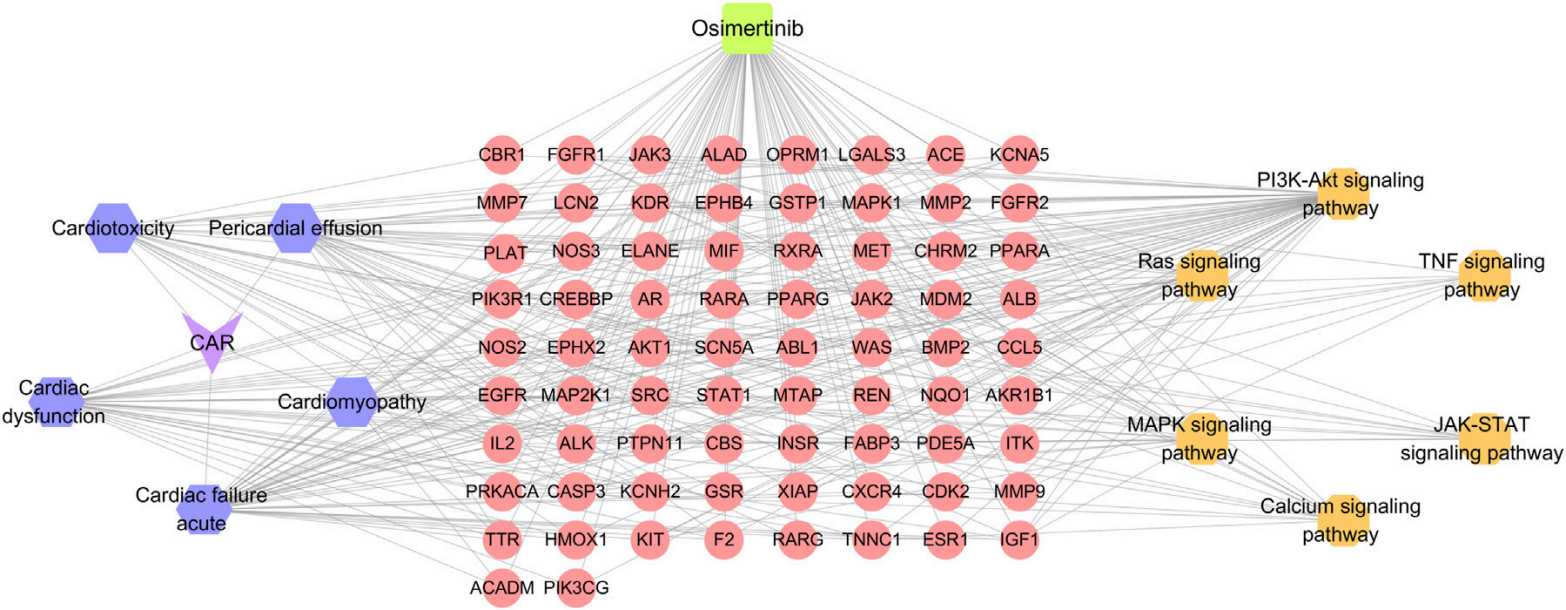
Drug-targets-CAR-pathways network. Purple for adverse reactions, orange for pathway, pink for core target, green for drug.

The drug-targets-CAR-pathways network was constructed using Cytoscape 3.7.2 software (Figure 2). The entire network consists of 87 nodes and 322 edges. Pink color denotes key targets, yellow represents pathways, purple signifies primary adverse cardiac reactions, and green indicates drug. Node rank value in the network reflects its connectivity to other nodes, with higher values indicating greater importance within the network. The pathways with high degree values were ras signaling pathway, calcium signaling pathway, MAPK signaling pathway. These pathways are considered to be key mechanisms underlying the CAR induced by osimertinib.

### 3.3 Molecular docking analysis

**FIGURE 3:**
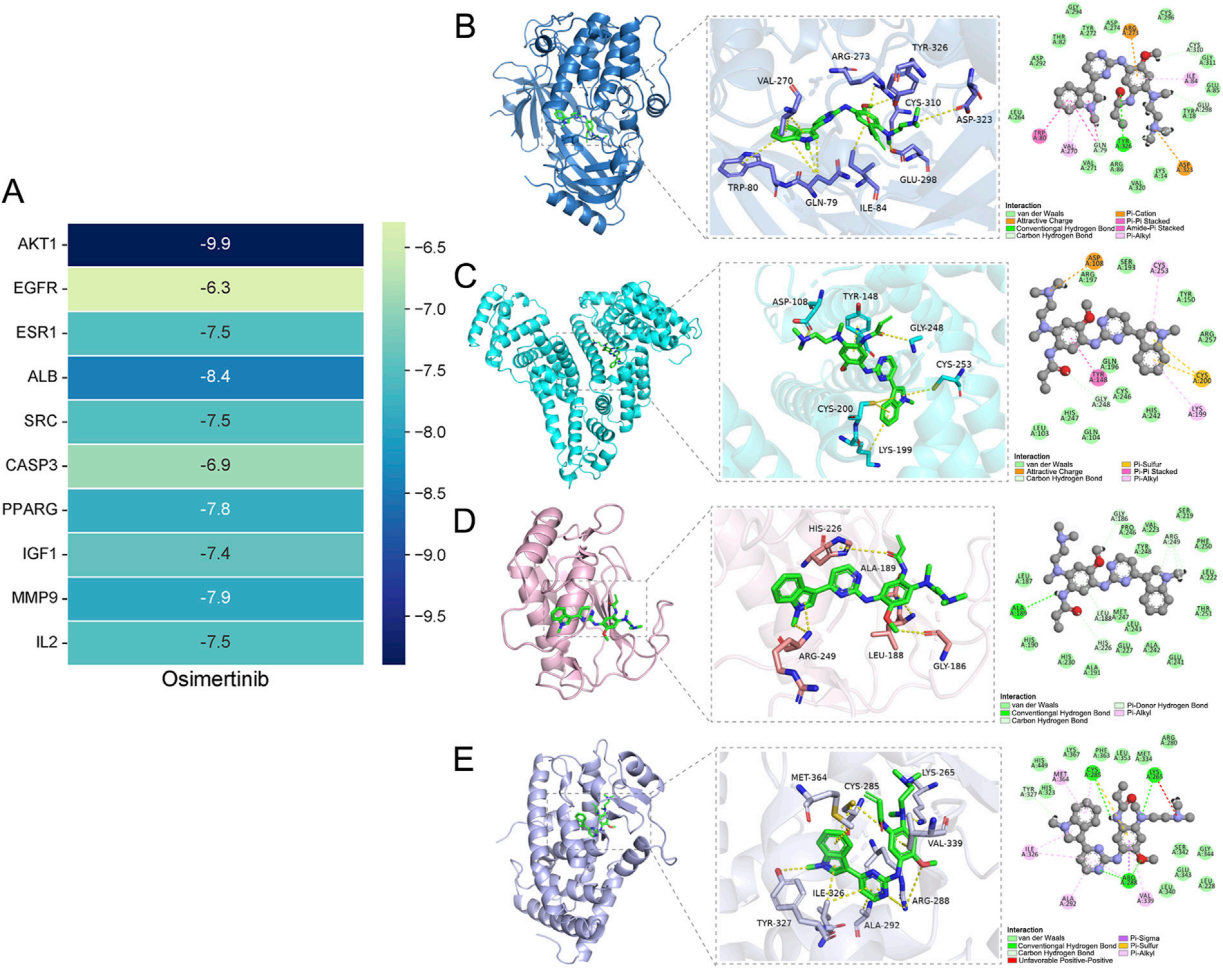
**(A)** Heatmap representation of molecular docking results. Molecular docking between the osimertinib and core targets. **(B)** The binding mode of AKT1 and osimertinib. **(C)** The binding mode of ALB and osimertinib. **(D)** The binding mode of MMP9 and osimertinib. **(E)** The binding mode of PPARG and Osimertinib.

The top 10 key targets (AKT1, ESR1, EGFR, SRC, ALB, CASP3, PPARG, MMP9, IGF1, IL2) and osimertinib were selected from the network for further process. Subsequently, docking analysis was performed using Chimera 1.16 vina. The binding energy serves as an indicator for assessing the spontaneous formation of stable structures between molecules. A prerequisite for the spontaneous binding is that the binding energy should be negative. The lower the value of the minimum binding energy, the stronger the affinity between the target protein and compound, leading to a more favorable formation of a stable structure. When the binding energy falls below −5.0 kJ/mol, it indicates good binding activity (Jia et al., 2021; Qasim et al., 2023; Zhan et al., 2023; Choi et al., 2024). The docking results were presented in Figure 3A. The binding energies of all core targets to the osimertinib were found to be less than −5 kJ/mol. Three-dimensional molecular docking results revealed that osimertinib exhibited strong binding affinity towards the core targets associated with adverse reactions of the heart. The PyMOL software was utilized to analyze and plot the docking results with a binding energy lower than −7.5 kJ/mol (Figures 3B–E). Among these interactions, osimertinib forms hydrogen bonds with the Tyr326 residue on the AKT1 protein receptor, exhibits carbon-hydrogen interactions with Glu298, Gln79, Tyr326, and Cys310 residues, and demonstrates hydrophobic interactions with Ile84, Val270, Trp80, and Gln79 residues. Additionally, osimertinib shows hydrophobic interactions with Cys253, Lys199, and Tyr148 residues on the ALB protein receptor, carbon-hydrogen interactions with Gly248, electrostatic interactions with Asp108, and Pi-Sulfur interactions with Cys200. In its interaction with the MMP9 protein receptor, osimertinib forms hydrogen bonds with Ala189 and exhibits carbon-hydrogen interactions with Gly186, Arg249, His226, and Leu188 residues. For the PPARG protein receptor, osimertinib forms hydrogen bonds with Cys285, Lys265, and Arg288 residues, demonstrates hydrophobic interactions with Met364, Ile326, Ala292, Val339, and Arg288 residues, and additionally exhibits Pi-Sigma interactions with Arg288 and Pi-Sulfur interactions with Cys285.

### 3.4 Molecular dynamics simulations

**FIGURE 4:**
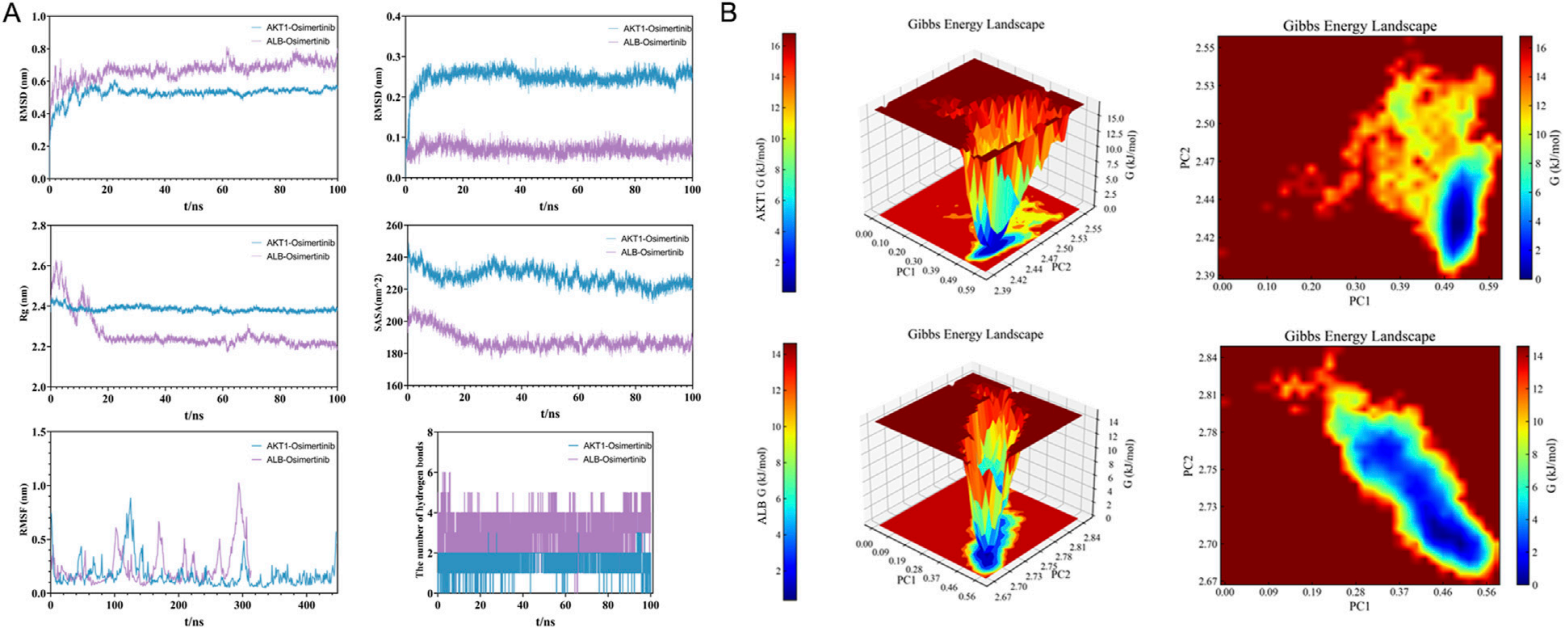
**(A)** 100 ns molecular dynamics simulation analysis of AKT1/ALB complex. **(B)** Gibbs free energy analysis of osimertinib–AKT1 and osimertinib–ALB complexes.

In this study, a 100-nanosecond molecular dynamics (MD) simulation was conducted on the osimertinib - AKT1/albumin complex system. The analysis encompassed root-mean-square deviation (RMSD), root-mean-square fluctuation (RMSF), radius of gyration (Rg), solvent-accessible surface area (SASA), and the statistical changes in hydrogen bonding throughout the simulation process. As illustrated in Figure 4A, the RMSD curve of the osimertinib-AKT1 complex exhibited a rapid increase during the initial 20 ns, followed by stabilization within the range of 0.4–0.6 nm, with an average RMSD value of 0.52 nm. Similarly, the RMSD curve of the osimertinib-ALB complex stabilized within the same range, with an average RMSD of 0.5 nm. The Rg of the osimertinib-AKT1 complex initially reached approximately 2.44 nm and subsequently stabilized at around 2.38 nm after 20 ns, indicating good compactness of the complex. In contrast, the Rg of the osimertinib-ALB complex started at 2.85 nm and gradually decreased to stabilize at approximately 2.7 nm after 20 ns.

Furthermore, SASA analysis revealed that the SASA curve of the osimertinib-AKT1 complex stabilized at around 220 nm^2^, decreasing from an initial value of 240 nm^2^. For the osimertinib-ALB complex, the SASA curve initially measured 330 nm^2^ and gradually decreased to stabilize at approximately 290 nm^2^ after 20 ns. These results suggest that the binding of osimertinib reduces solvent exposure for both complexes. RMSF analysis demonstrated that most amino acid residues in AKT1 exhibited relatively low fluctuation amplitudes, reflecting reduced flexibility and overall stability of the complex. In the case of ALB, most residues displayed low RMSF values, with only minor fluctuations observed at the N-terminal and C-terminal regions. Additionally, the number of hydrogen bonds formed between osimertinib and its respective protein partners varied within the range of 1–3 during the 100 ns simulation period.

To further elucidate the binding stability of the osimertinib-AKT1/ALB complexes, the MM/PBSA method was employed to calculate the binding free energy based on the final 10 ns stable RMSD trajectory. As presented in Table 4, the total binding free energy of the osimertinib-AKT1 complex was −44.63 kJ/mol, comprising contributions from van der Waals forces (−44.63 kJ/mol), electrostatic interactions (−21.90 kJ/mol), and gas-phase energy (−81.09 kJ/mol). These findings indicate favorable stability of the osimertinib-AKT1 complex system. Similarly, the osimertinib-ALB complex exhibited a total binding free energy of −42.92 kJ/mol, with contributions from van der Waals forces (−60.67 kJ/mol), electrostatic interactions (−5.32 kJ/mol), and gas-phase energy (−65.99 kJ/mol), supporting the stability of the osimertinib-ALB complex system.

**TABLE 4:** Binding free energy of osimertinib -AKT1/ALB complex.

Contribution components	AKT1-Osimertinib	ALB-Osimertinib
Δ_VDWAALS_	−59.19 ± 0.32	−60.67 ± 1.43
ΔE_elec_	−21.90 ± 1.43	−5.32 ± 2.07
ΔE_GB_	44.24 ± 1.76	30.03 ± 0.62
ΔE_surf_	−7.79 ± 0.06	−6.96 ± 0.03
ΔG_gas_	−81.09 ± 1.47	−65.99 ± 2.52
ΔG_solvation_	36.46 ± 1.76	23.08 ± 0.62
ΔTotal	−44.63 ± 2.29	−42.92 ± 2.59

The Gibbs free energy was calculated based on the RMSD and Rg values of the osimertinib-AKT1/ALB complexes, and corresponding 3D and 2D topography maps were generated using these parameters. As shown in Figure 4B, the Gibbs free energy topography maps for both complexes featured a single, sharp minimum energy region, confirming their thermodynamic stability.

## 4 Discussion

This study, based on the FAERS database, identified a total of 274 cardiac ADR reports associated with osimertinib. The results indicated a higher proportion among individuals aged 60 to 80 and above. Previous studies have shown that risk factors for cardiotoxicity from anti-tumor drugs include advanced age, pre-existing heart disease, and concurrent use of other cardiotoxic medications. The risk of cardiotoxicity associated with EGFR-TKIs is particularly linked to patients’ cardiovascular history, and elderly individuals are more susceptible to cardiovascular diseases (Sadasivan et al., 2020). Therefore, special attention should be given to elderly patients receiving osimertinib treatment. It is important to note that age itself is an independent risk factor for heart disease. Additionally, this study found that the majority of reports were concentrated in females, which may be due to the higher risk of cardiac events in elderly women (Nguyen et al., 2018). It is also important to note that EGFR mutations are more prevalent in females, which may contribute to this observed pattern. The higher prevalence of EGFR mutations in female patients naturally leads to increased osimertinib usage in this population, potentially resulting in more cardiac ADR reports among female patients (Choi et al., 2010; Liu X. D. et al., 2024). According to the report source analysis, the highest number of major adverse cardiac reaction reports originated from Asia. This may be attributed to the higher prevalence of EGFR mutations in Asian patients with NSCLC compared to European and American populations (Lynch et al., 2004; Paez et al., 2004; Pao et al., 2004; Melosky et al., 2022; Tan and Tan, 2022). Osimertinib exhibited a significantly higher risk of pericardial effusion, cardiomyopathy, cardiotoxicity, acute heart failure, and cardiac dysfunction compared to all other drugs in the FAERS database. Cardiac-related adverse drug reactions (ADRs) are common toxicities associated with tyrosine kinase inhibitors (TKIs), affecting not only the first and second generations but also the third generation (Anand et al., 2019; Alhoshani et al., 2020). Among osimertinib-induced cardiac toxicities, cardiac dysfunction has the highest fatality rate, reaching 25.00%, followed by acute heart failure at 20.00%. Additionally, among patients with cardiac dysfunction, the incidence of life-threatening events is the highest, at 16.67%. Studies have shown that 4.4% of patients taking osimertinib develop cancer treatment-related cardiac insufficiency. Some patients’ cardiac function returned to normal after dose reduction, while others did not recover even after discontinuing Osimertinib (Kunimasa et al., 2021). According to disproportionality signal analysis, the reporting odds ratio (ROR) for cardiac dysfunction associated with osimertinib compared to all other drugs in the FAERS database increased to 6.02 (95% CI: 4.02–9). Although the number of reports is relatively small, the signal strength is high, indicating a strong correlation. This suggests that in clinical practice, cardiac monitoring should be promptly conducted for patients with cardiac risk factors and those who develop cardiac-related signs and symptoms during treatment.

Currently, the mechanism underlying osimertinib-induced CAR remains unclear. To elucidate the potential mechanisms of major CAR caused by osimertinib and to clarify its correlation with clinical detection indicators, this study conducted network pharmacology and molecular docking analyses in addition to FAERS database analysis. Network pharmacology identified 74 intersection targets between osimertinib and major CAR, including key targets such as AKT1, ESR1, EGFR, SRC, ALB, CASP3, PPARG, MMP9, IGF1, and IL2. These findings suggest that osimertinib may induce CAR by interacting with these core target genes. Research indicates that in the early stages of myocardial fibrosis, AKT1 activity significantly increases and plays a critical role in regulating cardiomyocyte apoptosis and metabolism (Walkowski et al., 2022). EGFR, also known as the ErbB1 receptor, exerts complex effects on various cellular behaviors associated with cardiovascular diseases. A study found that EGFR activation is linked to cardiac remodeling, and antisense inhibition of EGFR can prevent the development of left ventricular hypertrophy (Kagiyama et al., 2003). The ESR1 gene, also referred to as the estrogen receptor α (ERα) gene, encodes the estrogen receptor ERα. ERα is an important transcription factor that plays a crucial role in modulating estrogen receptor-related signaling pathways. In wild-type animal models of cardiac ischemia, ERα activation can significantly reduce infarct size, myocardial cell apoptosis, inflammation, and oxidative stress, induce vasodilation, and promote neovascularization (Puzianowska-Kuźnicka, 2012). Studies have shown that the expression and phosphorylation levels of SRC are elevated in the myocardial tissue of mice with angiotensin II-induced cardiac fibrosis (Zhong et al., 2022). Decreased ALB levels may lead to hypoproteinemia, resulting in edema and reduced blood volume, thereby exacerbating the cardiac burden. Research has explored the relationship between hypoproteinemia and the prognosis of heart failure patients, revealing that low ALB levels are significantly associated with decreased survival rates (Horwich et al., 2008). CASP3 is a key protease in the process of apoptosis; improper regulation of CASP3 may lead to excessive or insufficient cardiac cell apoptosis, thereby affecting cardiac function (Yang et al., 2013; Asadi et al., 2022). Peroxisome proliferator-activated receptor γ (PPARG), when activated by ligands, can inhibit ventricular hypertrophy, improve ventricular remodeling, and enhance cardiac function (Miki et al., 2013). The dynamic balance of the extracellular matrix is influenced by changes in the levels of MMPs and their inhibitors TIMPs. Among these core targets, MMP9, a member of the MMP family, can influence cellular inflammatory responses, apoptosis, and the growth and differentiation of new blood vessels. Changes in its expression play a significant role in cardiac structural alterations and functional (Wetzl et al., 2017). Notably, while our analysis revealed multiple targets associated with cardiac remodeling and function, we observed a relative absence of targets directly related to pericardial physiology (such as VEGF-A and VEGFR2). This finding may provide a molecular basis for understanding the differential signal strength observed in our FAERS analysis, where the reporting odds ratio for pericardial effusion (approximately 3) was lower than that for cardiomyopathy and acute cardiac failure (approximately 5).

Combining KEGG pathway enrichment analysis, it was found that the potential targets of osimertinib are primarily enriched in signaling pathways associated with adverse cardiac reactions, including the PI3K-Akt signaling pathway, Ras signaling pathway, MAPK signaling pathway, calcium signaling pathway, JAK-STAT signaling pathway, and TNF signaling pathway. Research has demonstrated that the PI3K/Akt pathway is one of the most critical signaling pathways for regulating cardiomyocyte survival and function. This pathway controls cardiomyocyte survival and function by modulating the cell cycle, apoptosis-related factors at different stages of apoptosis, proliferation, autophagy, and necrosis, thereby playing a key regulatory role in cardiomyocyte survival and programmed death (Liu et al., 2017; Ghafouri-Fard et al., 2021; Ghafouri-Fard et al., 2022). Proteins enriched in the MAPK signaling pathway are closely associated with apoptosis, and the effector proteins have been validated (Zhou et al., 2022). Intracellular calcium ions play a critical role in regulating heart function, while extracellular calcium ions can trigger the release of intracellular calcium ions, thereby stimulating myocardial cell contraction. Calcium ions influence heart function through multiple mechanisms, including modulating calcium ion channels and sodium-calcium exchangers, and re-supplying calcium ions to the myocardium via SERCA2a on the sarcoplasmic reticulum, promoting myocardial relaxation (Metwally et al., 2020). The Ras signaling pathway promotes myocardial cell growth and protein synthesis by activating the downstream MAPK/ERK pathway, leading to myocardial hypertrophy. Long-term myocardial hypertrophy may result in heart failure and arrhythmia (Heineke and Molkentin, 2006). The JAK-STAT signaling pathway significantly affects heart function by regulating processes such as inflammation, fibrosis, and apoptosis. Studies have shown that the JAK-STAT pathway is activated by cytokines (such as IL-6, TNF-α), which promote the expression of inflammatory mediators, leading to myocardial inflammation and injury. Chronic inflammation can cause cardiomyopathy and heart failure (Chen et al., 2023; Jia et al., 2024). Activation of the JAK-STAT pathway also stimulates fibroblast proliferation and collagen deposition, resulting in myocardial fibrosis (Magaye et al., 2020). TNF-α, an inflammatory mediator, promotes cardiac fibrosis by activating the NLRP3 inflammasome, providing a potential new target for heart failure (Yamaguchi et al., 2022). Molecular docking studies identified 10 key targets that interacted with osimertinib. The binding energies of all core targets were lower than −5 kJ/mol, suggesting that osimertinib exhibits strong binding affinity to the core targets associated with CAR. Notably, AKT1 (−9.9 kJ/mol) and ALB (−8.4 kJ/mol) demonstrated the strongest binding among the selected targets.

The stability of the osimertinib-AKT1/ALB complex system was assessed via a 100-nanosecond molecular dynamics (MD) simulation. The RMSD results demonstrated that the binding of osimertinib with AKT1/ALB was relatively stable throughout the simulation. The RMSF analysis revealed significant domain flexibility in the regions interacting with osimertinib within both complexes. The small Rg values indicated that the overall structure became more compact upon binding. The SASA analysis showed a reduction in solvent-accessible surface area after binding, suggesting enhanced hydrophobic interactions and stabilization. Additionally, numerous hydrogen bonds were observed between osimertinib and its respective protein partners during the simulation. The Gibbs free energy calculations revealed that both complexes formed a single, sharp minimum energy region, indicating the presence of a highly stable and compact conformation post-binding. Based on these comprehensive molecular dynamics simulation results, it can be concluded that the interaction between osimertinib and AKT1/ALB contributes significantly to the overall structural stability, internal stability, compactness, surface characteristics, and interaction strength of the protein complexes.

Although this study utilized network pharmacology and molecular docking to investigate the potential mechanisms underlying osimertinib-induced cardiotoxicity, the findings remain predominantly theoretical in nature due to the absence of experimental validation through *in vitro* or *in vivo* studies. Consequently, the reliability and Pathophysiological relevance of the proposed mechanisms warrant further exploration. Moreover, while a large number of adverse event reports were analyzed, the sample size for specific cardiac adverse reactions (e.g., only 24 cases of cardiac dysfunction) was relatively limited, which may have constrained the statistical power to detect significant associations. Additionally, the study focused exclusively on osimertinib-associated cardiotoxicity without incorporating comparative analyses with other EGFR tyrosine kinase inhibitors (EGFR-TKIs), thereby limiting the ability to evaluate the specificity of osimertinib’s cardiac risks. Future research can utilize multi-omics joint analysis, organoid models and clinical cohort tracking, etc., to deeply dissect the spatiotemporal specificity mechanism of osimertinib-induced cardiotoxicity and make direct comparisons with other EGFR inhibitors, providing a basis for precise intervention.

## 5 Conclusion

This study identified key cardiac adverse reactions (CARs) of osimertinib, including pericardial effusion and cardiomyopathy, through FAERS data mining, with strong signals for cardiac dysfunction (ROR = 6.02). Integrated network pharmacology and molecular dynamics suggest potential mechanisms involving AKT1, EGFR, and pathways like PI3K-Akt and MAPK. While further experimental validation is needed, these findings emphasize the need for cardiac monitoring in high-risk patients and provide a foundation for safer clinical use and future research on osimertinib’s cardiotoxicity.

## Data Availability

The raw data supporting the conclusions of this article will be made available by the authors, without undue reservation.
