# Supplementary material for: An integrated approach based on FDA adverse event reporting system, network pharmacology, molecular docking, and molecular dynamics simulation analysis to study the cardiac adverse reactions and mechanism of action of osimertinib
Source: Front Pharmacol. 2025 Jun 9;16:1619517. doi: 10.3389/fphar.2025.1619517 (PMC12183265; doi:10.3389/fphar.2025.1619517)
Supplement: Supplementary file 1 [file DataSheet1.pdf]

Supplementary Material

TABLE S1 All reporting of adverse events in the cardiac disorders from 2015 to 2024.

PT	Reports number	percentage/% (Cardiac ADR)	percentage/% (full database)	ROR (95%CI)
Pericardial effusion	59	21.53	0.38	3.90(3.02~5.04)
Cardiomyopathy	47	17.15	0.31	5.31(3.98~7.08)
Cardiotoxicity	42	15.33	0.27	5.03(3.71~6.83)
Cardiac failure acute	25	9.12	0.16	5.15(3.47~7.64)
Cardiac dysfunction	24	8.76	0.16	6.02(4.02~9.00)
Left ventricular dysfunction	17	6.20	0.11	4.1(2.54~6.61)
Cardiac tamponade	16	5.84	0.10	4.31(2.64~7.06)
Ventricular hypokinesia	11	4.01	0.07	5.77(3.18~10.46)
Left ventricular failure	10	3.65	0.07	4.68(2.51~8.72)
Myocardial injury	9	3.28	0.06	10.19(5.26~19.71)
Ventricular dysfunction	8	2.92	0.05	7.79(3.87~15.67)
Toxic cardiomyopathy	3	1.09	0.02	12.53(3.98~39.42)
Congestive cardiomyopathy	3	1.09	0.02	4.58(1.47~14.28)

TABLE S2 Calculate the fourfold table.

Types of drugs	Number of target ADR reports	The number of other ADR reports	Total
Target drug	a	b	a+b
Other drugs	c	d	c+d
Total	a+c	b+d	N=a+b+c+d

TABLE S3 Formulas and thresholds of ROR method and BCPNN method.

Method	Calculation formula	Threshold
ROR method	ROR $=\frac{a/c}{b/d}$	If a is greater than or equal to 3 and the lower limit of the 95% confidence interval exceeds 1, one signal will be gen

BCPNN method	95%CI=$e^{\text{lnROR}\pm1.96\sqrt{\frac{1}{a}+\frac{1}{b}+\frac{1}{c}+\frac{1}{d}}}$<br>IC=$\log_2\frac{a(a+b+c+d)}{(a+b)(a+c)}$<br>$\gamma=\gamma_{ij}=\frac{(N+\alpha)(N+\beta)}{(a+b+\alpha_i)(a+c+\beta_j)}$<br>E（IC）=$\log_2\frac{(a+\gamma_{ij})(N+\alpha)(N+\beta)}{(N+\gamma)(a+b+\alpha_i)(a+c+\beta_j)}$<br>V（IC）=$\left(\frac{1}{\ln 2}\right)^2\left[\frac{N-a+\gamma-\gamma_{ij}}{(a+\gamma_{ij})(1+N+\gamma)}+\frac{N-a-b+\alpha-\alpha_i}{(a+b+\alpha_i)(1+N+\alpha)}+\frac{N-a-c+\beta-\beta_j}{(a+c+\beta_j)(1+N+\beta)}\right]$<br>SD=$\sqrt{V（IC）}$<br>IC025=E（IC）-2SD	If a is greater than or equal to 3 and the lower bound of the 95% confidence interval exceeds 0, one signal will be generated.

In the 2x2 contingency table for signal detection: a, number of reports containing both the drug of interest and the adverse event of interest; b, number of reports containing the drug of interest but not the adverse event of interest; c, number of reports containing the adverse event of interest but not the drug of interest; d, number of reports containing neither the drug of interest nor the adverse event of interest. ROR: Reporting Odds Ratio; IC (Information Component): logarithmic measure of the disproportionality between observed and expected values; E(IC): expected value of the Information Component; V(IC): variance of the Information Component; SD: standard deviation of the IC; IC025: lower bound of the 95% confidence interval for IC; γ and γij: Dirichlet distribution parameters (set as γij = 1); αi, α, βj, and β: Beta distribution parameters (set as α = β = 2, βj = αi = 1).

TABLE S4 Information on potential targets.

No	Types of adverse cardiac reactions	Targets	Number
1	Pericardial effusion	EGFR, ALB, REN, ACE, F2, ALK, EPHB4, IL2, PTPN11, TTR, MMP9, ELANE, MMP2, PLAT, SCN5A, MAP2K1, XIAP, CCL5, CBS, MIF, MET, ITK, CASP3, KDR, CXCR4, IGF1, MMP7, MAPK1, MTAP, PPARA	30
2	Cardiomyopathy	TNNC1, TTR, PTPN11, KCNH2, CHRM2, MAP2K1, CBS, ACADM, KCNA5	10
3	Cardiotoxicity	KCNH2, RARG, CASP3, CBR1, SCN5A, NOS3, EPHX2, PIK3R1, GSTP1, AKR1B1, FABP3, OPRM1, RXRA, LGALS3, GSR, KCNA5, EGFR<br>MDM2, HMOX1, CDK2, IL2, NQO1	22
4	Cardiac failure acute	SCN5A, KCNH2, ACE, TTR, KIT, PTPN11, JAK2, ABL1, ALB, F2, REN, TNNC1,	41

		ELANE, FGFR1, CXCR4, EGFR, AKT1, CREBBP, MAP2K1, RARA, BMP2, JAK3, ESR1, NOS3, LCN2, IGF1, STAT1, KDR, MMP9, CBS, ALAD, FGFR2, KCNA5, SRC, MAPK1, IL2, MET, HMOX1, WAS, AR, PIK3R1	
5	Cardiac dysfunction	SCN5A, KCNH2, TNNC1, ACE, TTR, BMP2, NOS3, PPARG, PDE5A, CNA5, AKT1, PTPN11, ALB, IGF1, CBS, REN, ACADM, F2, MAP2K1, PRKACA, MMP9, NOS2, EGFR, MAPK1, INSR, PIK3CG, CASP3, HMOX1	28

TABLE S5 Abbreviations Used.

Abbreviation	Full Form
CAR	Cardiac Adverse Reactions
FAERS	FDA Adverse Event Reporting System
ROR	Reporting Odds Ratio
BCPNN	Bayesian Confidence Propagation Neural Network
PPI	Protein-Protein Interaction
GO	Gene Ontology
KEGG	Kyoto Encyclopedia of Genes and Genomes
EGFR	Epidermal Growth Factor Receptor
NSCLC	Non-Small Cell Lung Cancer
TKIs	Tyrosine Kinase Inhibitors
ADR	Adverse Drug Reaction
RMSD	Root-Mean-Square Deviation
RMSF	Root-Mean-Square Fluctuation
Rg	Radius of Gyration
SASA	Solvent-Accessible Surface Area
MM-PBSA	Molecular Mechanics/Poisson-Boltzmann Surface Area
MD	Molecular Dynamics
PI3K-Akt	Phosphoinositide 3-Kinase-Protein Kinase B
MAPK	Mitogen-Activated Protein Kinase
JAK-STAT	Janus Kinase-Signal Transducer and Activator of Transcription
TNF	Tumor Necrosis Factor
SMILES	Simplified Molecular Input Line Entry System
PDB	Protein Data Bank
NVT	Canonical Ensemble (Constant Number, Volume, and Temperature)
NPT	Isothermal-Isobaric Ensemble (Constant Number, Pressure, and Temperature)
GAFF	General Amber Force Field

RESP	Restrained Electrostatic Potential
SERCA2a	Sarcoplasmic/Endoplasmic Reticulum Calcium ATPase 2a
NLRP3	NOD-like Receptor Family Pyrin Domain Containing 3
IL-6	Interleukin-6
TNF- α	Tumor Necrosis Factor-alpha
TIMP	Tissue Inhibitor of Metalloproteinases
MMP	Matrix Metalloproteinase
ER α	Estrogen Receptor Alpha
CC	Cellular Component
MF	Molecular Function
BP	Biological Process
